# Recipients’ and Environmental Factors Affecting the Pregnancy Rates of a Large, Fresh In Vitro Fertilization-Embryo Transfer Program for Dairy Cows in a Commercial Herd in China

**DOI:** 10.3390/vetsci11090410

**Published:** 2024-09-05

**Authors:** Chengyun Xie, Cong Huang, Longgang Yan, Ruiqi Yao, Jinbang Xiao, Mingmao Yang, Huatao Chen, Keqiong Tang, Dong Zhou, Pengfei Lin, Aihua Wang, Yaping Jin

**Affiliations:** 1 Key Laboratory of Animal Biotechnology of the Ministry of Agriculture, College of Veterinary Medicine, Northwest A&F University, Xianyang 712100, China; xiechengyun@nwafu.edu.cn (C.X.); cddhuangcong@163.com (C.H.); yanlonggang@nwafu.edu.cn (L.Y.); rachelyao157@gmail.com (R.Y.); xjb15680830507@yeah.net (J.X.); mingmao_yang@163.com (M.Y.); htchen@nwafu.edu.cn (H.C.); tangkeqiong20036@163.com (K.T.); zhoudong1949@163.com (D.Z.); linpengfei@nwafu.edu.cn (P.L.); wangaihua@nwafu.edu.cn (A.W.); 2 Department of Clinical Veterinary Medicine, College of Veterinary Medicine, Northwest A&F University, Xianyang 712100, China; 3 Department of Preventive Veterinary Medicine, College of Veterinary Medicine, Northwest A&F University, Xianyang 712100, China

**Keywords:** embryo transfer, pregnancy rate, dairy cows, ovum pick-up, in vitro fertilization, fresh embryos, China

## Abstract

**Simple Summary:**

The main objective of this study was to determine the influence of the recipient dairy cows’ breed, lactation number, estrus condition, the type, location and volume of the corpus luteum (CL) and the time of year that the embryo transfer (ET) was performed on the pregnancy rates of a large, fresh in vitro fertilization–embryo transfer program for dairy cows in a commercial herd in China. Our results showed that heifers with a larger CL without liquid cavity in the center of the CL and ET in spring significantly enhanced the pregnancy rates in dairy cows under farm conditions in the northwest region of China. These results can help breeders to select the best recipient dairy cows and improve the efficiency of embryo transfer in China.

**Abstract:**

The main objective of this study was to determine the influence of the recipient dairy cows’ breed, lactation number, estrus condition, the type, location and volume of the corpus luteum (CL) and the time of year that the embryo transfer (ET) was performed on the pregnancy rates of a large, fresh in vitro fertilization–embryo transfer program for dairy cows in a commercial herd in China. The recipients were from a herd of dairy cows in Ningxia, a province in northwest China, and we statistically analyzed the data of 495 cows from 2021 to 2023. Cumulus oocyte complexes (COCS) were isolated from follicular fluid obtained through ovum pick-up (OPU) and oocytes were incubated 20–22 h for in vitro maturation (IVM). Embryos were obtained after 10–12 h of in vitro fertilization (IVF) and six days of in vitro culture (IVC). Embryos at the morula or blastocyst stage were transferred to randomly chosen recipients (n = 495). The influence of recipients’ breed (Holstein or other), recipients’ lactation number (heifers or cows), estrus type (natural or synchronized), CL type (homogeneous, CL_hom_ or cavitary, CL_cav_), CL side (left or right), volume of the CL and season of transfer (spring, autumn or winter) on pregnancy rates were determined. The pregnancy rates were analyzed by binomial logistic regression with IBM SPSS statistics software, version 26. Pregnancy rates after ET to Holstein cows and other breeds were 43.49% and 42.68%, respectively (*p* > 0.05). Regarding age, pregnancy rates were 45.56% for heifers and 30.77% for cows (*p* < 0.05). Pregnancy rates following ET during natural and synchronized estrus were 44.41% and 41.5%, respectively (*p* > 0.05). Pregnancy rates with a left- or right-side CL were 40.18% and 45.65%, respectively (*p* > 0.05). The pregnancy rates achieved with a CL_hom_ and CL_cav_ were 44.44% and 39.68%, respectively (*p* < 0.05). The rates obtained in spring, autumn and winter were 49.26%, 46.02% and 34.64%, respectively (*p* < 0.05). Moreover, it was found that pregnancy rates were higher in recipients with a CL volume measuring greater than 10 cm^3^ compared with those with a CL volume measuring less than 10 cm^3^ (*p* < 0.05). The comparisons showed that recipients’ breed, estrus type or side of the CL had no effect, but the recipients’ lactation number, ET season and the type and volume of the CL have significant effects on pregnancy rates during ET.

## 1. Introduction

The development and use of biotechnology is indispensable for increasing reproductive efficiency. In this context, in vitro embryo production (IVEP) is a useful tool for the selection and breeding of genetically superior animals [1]. Recently, there has been an exponential increase in the number of in vitro-produced embryos worldwide. According to the International Embryo Technology Society (IETS) in 2017, for the first time, the number of embryos produced worldwide in vitro was significantly higher than the number of embryos produced in vivo [2].

The ultrasonic-guided follicular aspiration technique for the collection of COCs, also known as OPU, was originally developed for assisted reproduction in humans [3] and adapted for use in cattle [4]. Repetitive oocyte collections using OPU allow for greater production of in vitro-derived embryos, and ultimately pregnancies, in a fixed period of time than can be accomplished by traditional embryo collection/transfer procedures [5].

It is well known that ET is a powerful tool for increasing the number of offspring of donor cows, thus improving reproductive efficiency. ET is widely used to improve reproductive performance, to increase the number of animals with desirable genetic characteristics and to create high-yielding herds [6,7]. It is generally accepted that many factors related to the recipients’ physical condition and the environment can affect the establishment of ET pregnancies [7,8]. Healthy recipients are vital for an embryo transfer program, as they will sustain the length of the pregnancy and directly affect the development of the embryo and the performance of the animal after birth [7]. Different lactating cows can have different physical conditions which will affect the pregnancy rate [9]. In ruminants, progesterone (P4) is produced by the CL and is essential for the establishment and maintenance of pregnancy throughout gestation [10], and the CL can also differ as to type and size [11,12,13,14]. Since the embryo is transferred into the ipsilateral horn of the uterus, studies have shown that a CL of at least 17 mm in diameter ensures a higher pregnancy rate and better pregnancy maintenance [15,16]. The temperature and humidity are different in each season, and the effect of variations in the temperature humidity index (THI) should be considered because the change in temperature and humidity may affect the reproductive cycle and reproductive success rate of animals and the pregnancy rate after ET [17]. Some studies showed that a five-unit increase in maximum THI on the day of transfer was associated with an 18% increase in the odds of early embryonic loss [18]. Therefore, the influence of THI should be considered when discussing the influence of seasons on the pregnancy rate of embryo transfer. There have been many reports showing that recipients’ breed [9], recipients’ lactation number [11,12], type of estrus [13], type [14] and volume [19,20,21] of CL and season of ET [17] can influence ET pregnancy rates.

So far, most studies of ET programs for dairy cows have been from European and American regions, with obvious differences in climate and animal management methods compared with China [11,22,23]. Many studies showed that ET results from different regions were not the same [9,24]; therefore, the direct reference to ET data from different regions is likely to be inaccurate in China. Moreover, the demand for ET in China keeps increasing, so it is very important to obtain accurate data. We conducted the present study to provide data and technical support for the development of ET programs in China.

## 2. Materials and Methods

### 2.1. Chemical Reagents and Ethical Statement 

All chemicals and media were purchased from IVF Bioscience (Cornwall, UK) unless otherwise stated. The animal experiments adhered to the Ethics on Animal Care guidelines for the use of animals in this study, and the protocol was reviewed and approved by the Experiment Center of Northwest A&F University (Approval No. 2021028).

### 2.2. Climate and Animals

The cows used in this study were obtained from a large dairy farm in Lingwu, Ningxia, China, where the climate was classified as mid-temperate continental (latitude 37°35′–38°21′, longitude 106°11′–106°52′ E) with a mean annual temperature of 10.4 °C (−26.9 to 38.1 °C). The average temperatures in spring, autumn and winter were 10.8 °C, 9.8 °C and −5.3 °C, respectively. The average annual rainfall was 192.3 mm (49.4 rainfall days per year) and the rainfall in spring, autumn and winter were 36 mm, 41 mm and 4 mm, respectively. The relative humidity (%) in spring, autumn and winter was 35.67, 61 and 52, respectively. Relevant climatic conditions were quantified by calculating the THI, and the method was as follows [25]:

*THI* = (1.8 × *Td* + 32) − (0.55 − 0.55 × *RH* × 0.01) × (1.8 × *Td* − 26)

where *Td* = dry bulb temperature (air temperature in °C) and *RH* = % relative humidity. The *THI* in spring, autumn and winter was 56.3, 51.5 and 31.5, respectively.

The standard total mixed rations (TMR) diet was formulated in accordance with the NRC (2001) and included mainly compound feed, silage corn, corn, cottonseed cake, beet pulp and fodder grasses. The cows were fed twice a day and milked three times a day with an average milk yield of 38 kg/cow. Fresh water was available ad libitum. All of the cows were healthy and had no diseases of the hooves, mammary glands or reproductive organs.

Oocyte donors were healthy cows selected on the basis of genetic merit. Approximately 400 healthy Holstein cows, 3–6 years of age and with body condition scores of 3–4 (scale, 1–5), were used. 

### 2.3. Oocyte Collection and In Vitro Production (IVP) of Embryos

Donors for OPU were stimulated by FSH (Sansheng Co., Ningbo, Zhejiang, China), and we performed OPU twice a week. The multiovulation protocol involved puncturing the follicles of donor cows with a diameter greater than 5 mm on the first day, followed by injecting 150 units of FSH every 12 h for 36 h. A total of three injections were administered, and the OPU was performed 24 h after the third injection of FSH. Prior to OPU, cows received caudal epidural anesthesia (5 mL of 2% lidocaine; Sansheng Co., Ningbo, Zhejiang, China). The rectum was emptied, and the vulva and the perineal areas were cleaned. Follicles with a diameter of 2–8 mm were directly extracted from the ovaries of live Holstein cows using an ultrasonic scanner (Exapad-Mini, veterinary ultrasound scanner, IMV Co., Laegeler, France); we used OPU medium to wash the OPU system before and after operating. The needle used was 1.20 × 75 mm, and the vacuum pump was a model FV-6 (Fujihira Industry Co., Ltd., Tokyo, Japan) with a pressure of 70 mm Hg. The collected follicular fluid was transported to the laboratory near the farm at constant temperature within 10 min. The COCs were collected from follicular fluid with the aid of a stereo microscope, washed with HEPES-IVM medium in turn and incubated at 38.5 °C in 5% CO_2_ and 7% O_2_ with saturated humidity for 20–22 h. The oocytes were transferred to IVF medium, and washed Holstein spermatozoa (purchased from Pinyuan Co., Shijiazhuang, Hebei, China) were added to the oocytes at 1–6 × 10^6^ per mL. After fertilization, they were cultured for 10–12 h at 38.5 °C in 5% CO_2_ and 7% O_2_ with saturated humidity. The fertilized oocytes were transferred to SOF medium to remove granulosa cells and then transferred into IVC medium and cultured at 38.5 °C in 5% CO_2_ and 7% O_2_ with saturated humidity for three days. The IVC medium was replaced with fresh medium and incubation was continued until the seventh day after OPU, with the evaluation of developmental stage and quality of embryos (grade A to D) according to the standards of the International Embryo Technology Society [20]. Embryos were loaded into mini straws (IMV Co., France) in the form of transfer medium–air–transfer medium–air–embryo in transfer medium–air–transfer medium–air–transfer medium. The mini straws were then placed into the ET gun (IMV Co., France) for transfer stochastically. Only fresh embryos of grade A or B in the morula or blastocyst stage were transferred. 

### 2.4. Recipient Cows 

We selected healthy recipients with body condition scores of 3–4 (scale, 1–5), 60–150 days in milk, 40–45 kg for daily milk yield, with one lactation number who had no more than three matings. As for heifers, they were more than 14 months old, weighed more than 375 kg, had a body height of more than 130 cm, had good development with no abnormal body shape, were observed to have had at least two estrus cycles and were in estrus cycles with no more than three matings. All ETs to the recipients were randomly performed by a single experienced technician. Fresh ET requires that the embryos and the recipients maintain a synchronous physiological state. On day 1 after OPU, the oocytes were fertilized in vitro, and the selected recipients should be on the day of estrus. At this time, tail paint was used to make a preliminary estrus judgment of the selected recipients, and the cows’ external genitalia were severely congested, exhibiting redness and swelling, with the secretion of a large amount of mucus, and the cows accepted mounting from other cows. Finally, a B-mode veterinary ultrasound scanner (Easi-Scan, IMV imaging, Bellshill, UK) was used to determine estrus. The diameter of follicles in the ovary was measured and recorded, and cows with follicles approximately 2 cm (1.5–2.5 cm) in diameter were considered to be in estrus. On the second day after estrus, the ovaries of the recipients were checked again and, at this time, the follicles should have ovulated. If the cows had not ovulated, they were rejected as recipients. When cows had ovulated successfully, a functional CL would later be formed in the ovary, and the CL should be regular in shape and clear in boundary. On day 6 after OPU, the candidate cows were again examined by the ultrasound scanner, and they were selected as recipients for ET if the ovaries contained functional CL over 1 cm. A CL without liquid cavity in the center of the CL was classified as a CL_hom_. A CL with liquid cavity in the center of the CL was classified as a CL_cav_, and the diameter of this CL included the diameter of the liquid cavity. The following formula was used to calculate CL volume in cm^3^:

$$V_{\mathrm{CL}}=\frac{4}{3}\pi (\mathrm{CL}\;\mathrm{radius}{)}^{3}-\frac{4}{3}\pi (\mathrm{central}\;\mathrm{lacuna}\;\mathrm{radius}{)}^{3}$$

The recipients were divided according to estrus type into natural estrus and synchronized estrus. Because the ET program was for dairy cows in a commercial herd, the farm uniformly adopted the following estrus synchronization procedure for cows that had not been detected in estrus: GnRH was injected at D0, PGF2α was injected at D7, GnRH was injected at D9 and estrus was detected at D10.

### 2.5. Embryo Transfer

Embryos were transported to the farm from the laboratory within 30 min at a constant temperature of 38.5 °C on the seventh day after OPU and transferred by a single experienced technician into the cranial third of the uterine horn ipsilateral to the ovary with a CL. At the time of embryo transfer, the cows were restrained with a neck clamp, and 5 mL of 2% lidocaine hydrochloride (Sansheng Co., Huzhou City, China) was injected into the epidural space for spinal anesthesia. All embryos were implanted through the cervix and transferred to the uterine horn ipsilateral to the ovary containing a functional corpus luteum. On the 33rd–35th days after embryo transfer, the pregnancy status of the cows (yes/no) was determined by rectal ultrasonography, based on the presence of an embryo in the uterus.

### 2.6. Statistical Analysis 

The pregnancy rates were analyzed by binomial logistic regression with SPSS statistics software (version 26, IBM Co., New York, NY, USA, 2019). The recipients’ breed (Holstein or others), lactation number (heifers or cows), estrus condition (natural or synchronized), the type of CL (CL_hom_ or CL_cav_), CL location (right or left) and volume of CL (<5, 5–10 or >10 cm^3^) and the time of year (spring, autumn or winter) that the ET was performed were assumed to be independent variables, while the condition of being pregnant or not was taken as a dependent variable. Differences were considered significant when *p* < 0.05, and odds ratio, 95% confidence interval and *p* value were used to analyze the results.

## 3. Results

### 3.1. Oocyte Collection and In Vitro-Produced (IVP) Embryos

**Figure 1:**
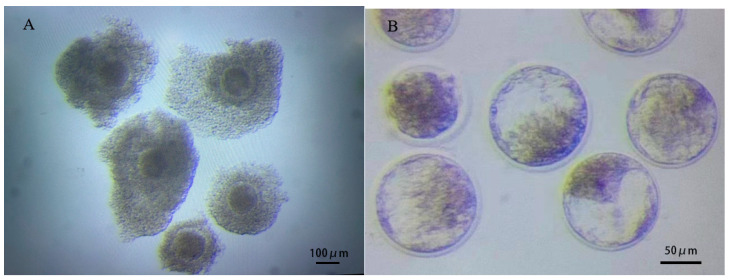
COCs and embryos at different culture stages during IVP: (**A**) COCs from OPU; (**B**) embryos obtained after IVM, IVF and IVC.

COCs (Figure 1A) were obtained from follicular fluid collected by OPU, and transferred embryos (Figure 1B) were obtained after IVM, IVF and IVC. OPU stimulated an average of 30–40 follicles at a time, accounting for nearly 70% of follicles of suitable size, and an average of 23 oocytes were obtained from each cow, with the proportion of Grade A and Grade B oocytes being >75%. The average oocyte recovery rate, cleavage rate and blastocyst rate of IVP from approximately 400 donor cows in this program were 72.80 ± 5.69%, 82.61 ± 2.69% and 40.18 ± 0.39%, respectively.

### 3.2. Embryo Transfer 

In this study, 495 embryos were transferred, and the overall pregnancy rate after ET was 43.23%. There was no significant difference in the pregnancy rate of recipients of different species, recipients’ estrus type or which side the CL was on (*p* > 0.05). However, the probability of pregnancy (odds ratio, OR) was higher following ET to Holstein recipients (OR = 1.249), with natural estrus (OR = 1.324) and the CL on the right side (OR = 1.199) (Table 1). 

Pregnancy rates following ET to heifers and cows were 45.56% and 30.77%, respectively (*p* < 0.05). Following the transfer to heifers, the OR was found to be 2.263 (Table 1). The type of CL had an effect on pregnancy rates (*p* < 0.05), and the rates achieved with a CL_hom_ and CL_cav_ were 44.44% and 39.68%, respectively. When ET was performed into the CL_hom_, the OR was found to be 1.616 (Table 1). In addition, it was found that pregnancy rates were higher in recipients with CL volumes >10 cm^3^ (61.04%) compared with those with CL volumes < 10 cm^3^ (*p* < 0.05), and the OR was lower following transfers when the CL volumes were 0–5 cm^3^ (39.56%; OR = 0.300) and 5–10 cm^3^ (43.14%; OR = 0.334) (Table 1). Moreover, we showed that transfers in spring (49.26%; *p* < 0.05; OR = 1.884) were more likely to result in pregnancies than those in winter (34.64%), although there was no difference in the pregnancy rate between autumn (46.02%; *p* > 0.05) and winter (OR = 1.616) (Table 1).

## 4. Discussion

There were millions of dairy cows with 40.27 million metric tons of milk produced in 2022 in China (http://food.china.com.cn/2023-07/21/content_93538997.htm, accessed on 15 January 2024), and there is a growing demand for more efficient embryo transfer in China. However, most studies about embryo transfer programs for dairy cows have been from European and American regions, with few reports on ET in China. Therefore, we have committed to promoting the development of embryo transfer in China. ET is the fastest way to change genetic traits for increased productivity in cattle. It is important to increase pregnancy success rates because of the greater expense and longer time needed for ET compared with artificial insemination. The selection of the best embryo recipients during ET is one of the crucial points for ensuring the success of the procedure [7,26].

In this study, 495 fresh IVP embryos were obtained through OPU, IVM, IVF and IVC and transferred. To minimize variations due to the animals, all the cows were healthy, in similar physical condition and subject to exactly the same procedures. Many factors, such as breed, nutrition and management, are involved in a successful embryo transfer. We found no significant differences in pregnancy rates after ET with respect to recipients’ breed, estrus type or side of the CL under the conditions of a farm in northwest China, and the results were similar to many reports all over the world [9,11,23]. 

In our research, we found that recipients’ lactation number and type and volume of the CL have statistically significant effects on pregnancy rates; in other studies, differences in the recipient cows also affected the pregnancy rate after ET [27,28,29]. We observed that when the recipients were heifers, the pregnancy rate was higher. This is in agreement with previous studies that showed that younger cows were better able to regulate body temperature as ambient temperature increased compared with older animals [29], and many studies showed that pregnancy rates decreased when body temperature increased [29,30]. Our results showed that the type of CL did have an impact on the pregnancy rates. Many veterinarians have doubts about the quality of the CL_cav_, because it is believed that the CL_cav_ cannot have the same function as the CL_hom_ [18,31]. However, some research results showed that there was no significant difference in the pregnancy rates between different types of CL [14], and this conclusion might have come about because of differences in feeding management and environment in the study area. Regarding the effect of the volume of the CL on pregnancy rates, our results agree with those of previous studies in which a larger CL resulted in higher pregnancy rates, and studies have shown that a CL of at least 17 mm in diameter ensures a higher pregnancy rate and better pregnancy maintenance [15,16,19,20,32]. This could be because larger CL structures maintain higher circulating progesterone concentrations in dioestrus according to previous reports [19,20,32]. There is also a positive correlation between plasma progesterone concentration and CL volume [18]. 

It has been shown that the seasons have a great influence on the efficiency of embryo transfer [9,33], especially in the summer when heat stress is a problem and pregnancy rate is the lowest [34,35,36]. Heat stress can compromise the reproductive events required for embryo development by decreasing expression of estrus behavior, altering follicular growth, compromising oocyte competence and inhibiting embryonic development, which can lead to pregnancy failure [1,36]. In addition, the temperature and humidity were different in different seasons, which led to the difference in THI, and some studies have shown that the change in THI affects the pregnancy rate, cleavage and embryo development [30,37]. Studies have shown that early embryonic loss was observed in 21% of embryo transfers, and a five-unit increase in maximum THI on the day of transfer was associated with an 18% increase in the odds of early embryonic loss [18]. We compared the pregnancy rates of ET in spring, autumn and winter, and the results showed that ET in spring was optimal for northwest China, possibly because the temperature and humidity in northwest China in spring were most suitable for cows.

To maximize pregnancy rates from embryo transfer, it is essential to rigorously control the conditions during actual production. More ETs should be carried out in suitable seasons, and the number of ETs should be reduced in seasons with low pregnancy rates. As far as possible, heifers should be selected as the recipients for embryo transfer, the recipients should be thoroughly examined before ET and those with a larger CL_hom_ should be selected.

## 5. Conclusions

Our results showed that heifers with a larger CL_hom_ and ET in spring significantly enhanced the pregnancy rates in dairy cows under farm conditions in the northwest region of China. However, recipients’ breed, estrus type or side of the CL were found to have no effect on pregnancy rates. These results can help breeders to select the best recipient dairy cows and improve the efficiency of embryo transfer in China.

## Figures and Tables

**Table 1:** Pregnancy rates on days 33–35 and odds ratios based on the recipients’ species, lactation number, estrus type, type of CL, side of CL, volume of CL and seasons.

Variable Categories	Pregnant	Not Pregnant	Total	Pregnancy Rate (%)	Odds Ratio	95% Confidence Interval	*p*
Species							
Holstein	147	191	338	43.49	1.249	0.758–2.056	>0.05
Others	67	90	157	42.68	Reference		
Lactation Number							
Heifers (0)	190	227	417	45.56	2.263	1.242–4.121	<0.05
Cows (1)	24	54	78	30.77	Reference		
Estrus Type							
Natural	131	164	295	44.41	1.324	0.82–2.137	>0.05
Synchronized	83	117	200	41.5	Reference		
CL Side							
Right	126	150	276	45.65	1.199	0.825–1.742	>0.05
Left	88	131	219	40.18	Reference		
CL Type							
CL_hom_	164	205	369	44.44	1.616	1.035–2.522	<0.05
CL_cav_	50	76	126	39.68	Reference		
CL Volume (cm^3^)							
<5	125	191	316	39.56	0.300	0.169–0.535	<0.05
5–10	44	58	102	43.14	0.334	0.172–0.648	<0.05
>10	47	30	77	61.04	Reference		
Season							
Spring	100	103	203	49.26	1.884	1.209–2.937	<0.05
Autumn	52	61	113	46.02	1.512	0.887–2.576	>0.05
Winter	62	117	179	34.64	Reference		

## Data Availability

The raw data used and/or analyzed during the current study are available from the corresponding author upon reasonable request.
